# Supplementary material for: Bos taurus and Cervus elaphus as Non-Seasonal/Seasonal Models for the Role of Melatonin Receptors in the Spermatozoon
Source: Int J Mol Sci. 2022 Jun 3;23(11):6284. doi: 10.3390/ijms23116284 (PMC9181011; doi:10.3390/ijms23116284)
Supplement: Supplementary file 1 [file ijms-23-06284-s001.zip › ijms-1708230-supplementary.pdf]

International Journal of *Molecular Sciences*

MDPI

Article

Bos taurus and *Cervus elaphus* as Non-Seasonal/Seasonal Models for the Role of Melatonin Receptors in the Spermatozoon

Estela Fernández-Alegre [1,2], Estíbaliz Lacalle [1,2], Cristina Soriano-Úbeda [1,3], José Ramiro González-Montaña [1,4], Juan Carlos Domínguez [1,4], Adriana Casao [5] and Felipe Martínez-Pastor [1,3,*]

[1] Institute of Animal Health and Cattle Development (INDEGSAL), University of León, 24071 Leon, Spain; efernandez@bianorbiotech.es (E.F.-A.); elacalle@bianorbiotech.es (E.L.); c.soriano.ubeda@unileon.es (C.S.-Ú.); jrgonm@unileon.es (J.R.G.-M.); jcdomt@unileon.es (J.C.D.)
[2] Bianor Biotech SL, 24071, Leon, Spain
[3] Department of Molecular Biology (Cell Biology), University of León, 24071, Leon, Spain
[4] Department of Medicine, Surgery and Veterinary Anatomy (Animal Medicine and Surgery), University of León, 24071, Leon, Spain
[5] Department of Biochemistry and Molecular and Cell Biology, Institute of Environmental Sciences of Aragón, School of Veterinary Medicine, University of Zaragoza, 50013, Zaragoza, Spain; adriana@unizar.es
* Correspondence: felipe.martinez@unileon.es; Tel.: +34-987-291-491

Citation: Fernández-Alegre, E.; Lacalle, E.; Soriano-Úbeda, C.; González-Montaña, R.; Domínguez, J.C.; Casao, A.; Martínez-Pastor, F. *Bos taurus* and *Cervus elaphus* as Non-Seasonal/Seasonal Models for the Role of Melatonin Receptors in the Spermatozoon. *Int. J. Mol. Sci.* **2022**, *23*, 6284. https://doi.org/10.3390/ijms23116284

Academic Editors: Elisabetta Baldi and Honoo Satake

Received: 16 April 2022
Accepted: 1 June 2022
Published: 3 June 2022

Publisher's Note: MDPI stays neutral with regard to jurisdictional claims in published maps and institutional affiliations.

Copyright: © 2022 by the authors. Licensee MDPI, Basel, Switzerland. This article is an open access article distributed under the terms and conditions of the Creative Commons Attribution (CC BY) license (http://creativecommons.org/licenses/by/4.0/).

Supplementary material

Tables S1 and S2 in this Supplementary Material show the parameters in the sperm samples after dilution at 50×10^6 ml^{-1} in TALP-HEPES and before incubation.

Table S1. Statistics for the sperm parameters in the initial assessment for bull.

Parameter	Lower hinge	Q1	Median	Q3	Higher hinge
Viability (PI-) (%)	57.2	68.6	75.3	78.3	87.2
Viability (YO-PRO-1-) (%)	62.5	67.3	72.5	75.7	81.3
Apoptotic (ratio viable) (%)	0.0	0.0	1.6	2.4	2.7
Reacted acrosome (%)	9.8	13.7	15.0	20.4	25.4
Active mitochondria (%)	58.5	64.9	72.2	74.9	78.4
Active mitochondria (ratio viable) (%)	95.6	97.1	98.3	98.8	99.1
Cytoplasmic ROS (mean) (MFI)	3.7	4.0	4.5	4.7	5.5
Mitochondrial ROS (ratio viable) (%)	3.8	4.1	4.3	4.5	5.0
Total motility (%)	69.7	73.0	75.4	85.2	90.9
Progressive motility (%)	33.9	38.0	42.1	46.5	53.1
Slow population (%)	0.0	7.2	9.4	13.5	15.2
Fast population (%)	0.0	0.0	0.0	28.3	57.1
Active population (%)	27.6	54.8	82.4	90.8	94.5
[Ca^{2+}] (mean) (MFI)	2.4	2.6	3.0	3.1	3.9
[Ca^{2+}] (mean) after ionophore (MFI)	4.1	4.8	5.9	6.4	6.9
[Ca^{2+}] (mean) after ionophore, ratio (%)	118.3	129.4	139.2	149.2	152.7
Capacitated (ratio viable) (%)	3.5	3.8	4.1	4.5	4.7
Capacitated (ratio viable) after LPC (%)	4.8	5.0	5.8	12.0	18.1
Capacitated (ratio viable) after LPC, ratio (%)	103.0	109.4	123.7	172.0	235.1
Reacted acrosome (ratio viable) (%)	0.2	0.2	0.3	0.4	0.6
Reacted acrosome (ratio viable) after ionophore (%)	0.3	0.3	0.4	0.6	0.6
Reacted acrosome (ratio viable) after ionophore, ratio (%)	75.9	96.3	106.4	134.5	152.8
Reacted acrosome (ratio viable) after LPC (%)	0.3	1.4	2.7	4.2	5.3
Reacted acrosome (ratio viable) after LPC, ratio (%)	102.8	199.8	298.9	373.8	450.7
F pattern (CTC), non-capacitated (%)	62.5	65.3	74.8	77.1	78.7
B pattern (CTC), capacitated (%)	10.9	13.6	18.7	22.7	25.7
Reacted acrosome (CTC) (%)	6.4	8.6	9.5	11.1	11.8

Q1 and Q3 are the first and third quartiles of the data distribution. The lower and upper hinge are the most extreme values within 1.5 times the interquartile range (minimum and maximum not considering extreme values —outliers). .

Table S2. Statistics for the sperm parameters in the initial assessment for red deer.

Parameter	Lower hinge	Q1	Median	Q3	Higher hinge
Viability (PI-) (%)	82.6	84.7	84.8	87.2	87.2
Viability (YO-PRO-1-) (%)	61.8	68.2	83.8	88.9	92.6
Apoptotic (ratio viable) (%)	0.0	0.0	1.1	25.1	28.1
Reacted acrosome (%)	7.9	7.9	8.7	9.7	10.7
Active mitochondria (%)	81.5	82.2	85.3	85.6	90.1
Active mitochondria (ratio viable) (%)	98.8	98.8	99.1	99.4	99.4
Cytoplasmic ROS (mean) (MFI)	2.8	3.2	3.9	3.9	4.5
Mitochondrial ROS (ratio viable) (%)	3.4	3.5	3.7	4.4	5.0
Total motility (%)	70.7	80.5	89.6	94.1	95.4
Progressive motility (%)	26.5	29.2	50.2	55.0	56.1
Slow population (%)	6.2	8.6	18.3	25.9	27.6
Fast population (%)	0.0	0.0	0.0	0.0	0.0
Active population (%)	72.3	74.2	81.7	91.4	93.8
[Ca^{2+}] (mean) (MFI)	2.7	3.3	4.5	4.5	5.7
[Ca^{2+}] (mean) after ionophore (MFI)	4.8	5.7	6.4	8.8	13.1
[Ca^{2+}] (mean) after ionophore, ratio (%)	103.3	112.8	153.6	154.9	164.9
Capacitated (ratio viable) (%)	3.2	3.7	4.1	5.5	5.5
Capacitated (ratio viable) after LPC (%)	3.2	4.4	7.5	10.3	10.3
Capacitated (ratio viable) after LPC, ratio (%)	76.0	116.7	136.9	169.8	210.2
Reacted acrosome (ratio viable) (%)	0.1	0.3	0.5	0.7	0.7
Reacted acrosome (ratio viable) after ionophore (%)	0.1	0.4	0.5	0.6	0.6
Reacted acrosome (ratio viable) after ionophore, ratio (%)	71.2	109.0	120.3	153.3	153.3
Reacted acrosome (ratio viable) after LPC (%)	0.7	0.8	1.1	1.1	1.1
Reacted acrosome (ratio viable) after LPC, ratio (%)	94.8	128.2	151.5	187.6	187.6
F pattern (CTC), non-capacitated (%)	78.8	80.8	82.4	86.5	87.1
B pattern (CTC), capacitated (%)	7.4	9.3	9.6	11.5	11.5
Reacted acrosome (CTC) (%)	3.6	3.9	4.2	7.7	10.2

Q1 and Q3 are the first and third quartiles of the data distribution. The lower and upper hinge are the most extreme values within 1.5 times the interquartile range (minimum and maximum not considering extreme values —outliers). .
